# Author Correction: Realization of Lieb lattice in covalent-organic frameworks with tunable topology and magnetism

**DOI:** 10.1038/s41467-020-14513-8

**Published:** 2020-01-29

**Authors:** Bin Cui, Xingwen Zheng, Jianfeng Wang, Desheng Liu, Shijie Xie, Bing Huang

**Affiliations:** 1 School of Physics, National Demonstration Center for Experimental Physics Education, Shandong University, Jinan, 250100 China; 2 Beijing Computational Science Research Center, Beijing, 100193 China

**Keywords:** Density functional theory, Ferromagnetism, Molecular electronics, Spintronics

Correction to: *Nature Communications* 10.1038/s41467-019-13794-y, published online 2 January 2020.

The original version of this Article contained an error in the first sentence of the Acknowledgements, which incorrectly listed the funding number as ‘11574188’. The correct version of the funding number is ‘11574118’.

This has been corrected in both the PDF and HTML versions of the Article.

