# Supplementary material for: Realization of Lieb lattice in covalent-organic frameworks with tunable topology and magnetism
Source: Nat Commun. 2020 Jan 2;11:66. doi: 10.1038/s41467-019-13794-y (PMC6940388; doi:10.1038/s41467-019-13794-y)
Supplement: Supplementary file 1 — Supplementary Information [file 41467_2019_13794_MOESM1_ESM.pdf]

Supplementary Information for
Realization of Lieb Lattice in Covalent-organic Frameworks with Tunable Topology and Magnetism
by Cui *et al.*

This PDF file includes:

Supplementary Note 1. Tight-binding (TB) Calculations for an Ideal Lieb Lattice.

Supplementary Note 2. TB Calculations for Distorted Lieb Lattice with Zeeman and spin-orbit coupling Effects.

Supplementary Note 3. TB calculations for distorted Lieb lattice with SOC effect.

Supplementary Note 4. Chern number and Berry curvature calculations.

Supplementary Note 5. Edge States Calculations.

Supplementary Note 6. Molecular-ligand-based Lieb lattice.

Supplementary Note 7. Six-band tight-binding modeling.

Supplementary Note 8. Half-metallic Semimetal.

Supplementary Figures 1-10.

Supplemantary Table 1.

Supplementary Information for Realization of Lieb Lattice in Covalent-organic Frameworks with Tunable Topology and Magnetism

Bin Cui[1,*], Xingwen Zheng[1], Jianfeng Wang[2], Desheng Liu[1], Shijie Xie[1], & Bing Huang[2,*]

[1]School of Physics, National Demonstration Center for Experimental Physics Education, Shandong University, Jinan 250100, China
[2] Beijing Computational Science Research Center, Beijing 100193, China

*Correspondence to B.C. (email: cuibin@sdu.edu.cn) or to B.H. (email: bing.huang@csrc.ac.cn).

Supplementary Note 1. Tight-binding (TB) Calculations for an Ideal Lieb Lattice.

$$H_0 = \begin{pmatrix} dE & -2t_1\cos(k_x a/2) & -2t_1\cos(k_y a/2) \\ -2t_1^*\cos(k_x a/2) & 0 & 0 \\ -2t_1^*\cos(k_y a/2) & 0 & 0 \end{pmatrix} \tag{1}$$

At the corner of BZ, e.g., M$\left(\frac{\pi}{a},\frac{\pi}{a}\right)$, $dk_x = k_x - \frac{\pi}{a}$ and $dk_y = k_y - \frac{\pi}{a}$

$$\varepsilon_2 = 0 \ \text{(the flat band)}$$

$$\varepsilon_{1,3} = \frac{dE \pm \sqrt{dE^2 + 16t_1t_1^*[\sin^2(dk_x a/2) + \sin^2(dk_y a/2)]}}{2}$$

At M point $\left(\frac{\pi}{a},\frac{\pi}{a}\right)$, and $dk_x \to 0, dk_y \to 0$,

$$\sin^2(dk_x a/2) + \sin^2(dk_y a/2) \to (dk_x a/2)^2 + (dk_y a/2)^2$$

$$\varepsilon_{1,3} = \frac{dE \pm \sqrt{dE^2 + t_1t_1^*({dk_x}^2 + {dk_y}^2)\frac{a^2}{2}}}{2}$$

When $dE = 0$,

$$\varepsilon_{1,3} = \frac{\pm\sqrt{2t_1t_1^*({dk_x}^2 + {dk_y}^2)}}{4} a \approx \pm\frac{\sqrt{2}a}{4}|t_1|dk$$

Therefore, $\varepsilon_{1,3}$ have linear dispersions near the M point, and form a Dirac cone.

When $dE \neq 0$ ($dE > 0$), the Dirac cone is gapped at M($\frac{\pi}{a}, \frac{\pi}{a}$) [or M1($\frac{\pi}{a}, -\frac{\pi}{a}$)],

$$\varepsilon_1 = \frac{dE + \sqrt{dE^2 + t_1 t_1^* \left(dk_x{}^2 + dk_y{}^2\right)\frac{a^2}{2}}}{2} = dE, \text{and } \varepsilon_3 = 0$$

Therefore, at the BZ corners, the middle flat band and the bottom Dirac band keep touching with each other, and a gap of $\Delta_1 = \varepsilon_1 - \varepsilon_2 = dE$ is opened between the top Dirac band and the lower two bands.

Supplementary Note 2. TB Calculations for Distorted Lieb Lattice with Zeeman and spin-orbit coupling Effects.

The total Hamiltonian with Zeeman and spin-orbit coupling (SOC) effects can be rewritten as:

$$H = H_{0+\mathrm{M}} + H_{\mathrm{so}} \tag{2}$$

We rewrite the Hamiltonian of Supplementary Equation 1 according to the coordination depicted in Fig. 1 in the main text, i.e., $a_1 = a(\sin\frac{\theta}{2}, \cos\frac{\theta}{2})$ and $a_2 = a(\sin\frac{\theta}{2}, -\cos\frac{\theta}{2})$, and the reciprocal lattice vectors are $b_1 = \frac{\pi}{a}(\csc\frac{\theta}{2}, \sec\frac{\theta}{2})$ and $b_2 = \frac{\pi}{a}(-\csc\frac{\theta}{2}, \sec\frac{\theta}{2})$. Because $\theta \neq \frac{\pi}{2}$, we use the (k_1, k_2) to denote a general **k** point. The rotated Supplementary Equation 1 with NNN hopping is:

$$H_0 = \begin{pmatrix} E_1 & E_{12} & E_{13} \\ E_{21} & E_2 & E_{23} \\ E_{31} & E_{32} & E_3 \end{pmatrix}, \tag{3}$$

where, $E_1 = dE/2$ and $E_2 = E_3 = dE/2$. The NN hoppings are $E_{12} = -2t_1\cos(k_1 a/2)$, $E_{21} = E_{12}^*$, $E_{13} = -2t_1\cos(k_2 a/2)$ and $E_{31} = E_{13}^*$, and the NNN hoppings are $E_{23} = -2t_2\cos[(-k_1 + k_2)\,a/2] - 2t_3\cos[(k_1 + k_2)\,a/2]$ and $E_{32} = E_{23}^*$.

When a Zeeman term M_{zi} is induced, the H_0 is extended to 6×6 as $H_{0+\mathrm{M}}$,

$$H_{0+\mathrm{M}} = H_0 \otimes I_2 + \begin{pmatrix} M_{z1} & 0 & 0 \\ 0 & M_{z2} & 0 \\ 0 & 0 & M_{z3} \end{pmatrix} \otimes \sigma_z.$$

where I_2 and σ_z are the 2×2 identity matrix and the Pauli matrix, respectively.

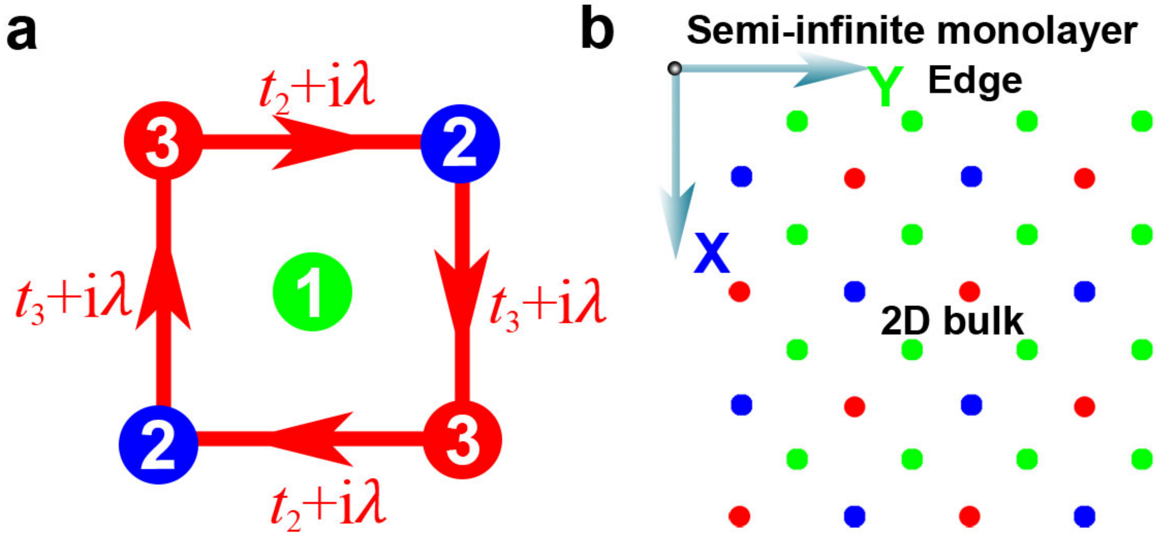

Supplementary Figure 1 | Schematic graphs of **(a)** the Kane and Mele's SOC term and **(b)** the semi-infinite Lieb lattice with an edge termination of site 1.

The Kane-Mele SOC term[1,2] is added between the NNN sites as an imaginary hopping, as shown in Supplementary Figure 1**a**, where the red arrows indicates the positive signs:

$$H_{\mathrm{SO}} = -i\lambda \sum_{\langle\langle i,j\rangle\rangle} (\mathbf{d}_{ik} \times \mathbf{d}_{kj}) \cdot \mathbf{s}^{z}_{\alpha\beta} c^{\dagger}_{i\alpha} c_{j\beta} \tag{4}$$

where λ is the SOC constant and $\mathbf{d}_{ik}$ ($\mathbf{d}_{kj}$) denotes the unit vector from edge (corner) site i (k) to corner (edge) site k (j) (see Supplementary Figure 1**a**). **s** is the Pauli matrix representing the electron spin. Since the SOC hopping is small, we can safely neglect the distortion effect and choose a homogeneous strength of the SOC along each direction. Therefore, the SOC term can be determined by:

$$H_{\mathrm{so}} = \begin{pmatrix} 0 & 0 & 0 & & & \\ 0 & 0 & E_{\mathrm{so}} & & 0 & \\ 0 & E^{*}_{\mathrm{so}} & 0 & & & \\ & & & 0 & 0 & 0 \\ & 0 & & 0 & 0 & E^{*}_{\mathrm{so}} \\ & & & 0 & E_{\mathrm{so}} & 0 \end{pmatrix}$$

where the $E_{\mathrm{so}} = -2\mathrm{i}\lambda\cos[(-k_1 + k_2)\,a/2] + 2\mathrm{i}\lambda\cos[(k_1 + k_2)\,a/2]$

Finally, the total Hamiltonian, i.e., Supplementary Equation 2, is diagonalized to obtain the energy bands and the Bloch states.

Supplementary Note 3. TB calculations for distorted Lieb lattice with SOC effect.

Taking $\mathrm{M1}(\frac{\pi}{a}, -\frac{\pi}{a})$ at the BZ corner as an example, the Hamiltonian can be written as

$$H(\mathrm{M1}) = \begin{pmatrix} dE & 0 & 0 \\ 0 & 0 & 2(t_2 - t_3) - 4\mathrm{i}\lambda \\ 0 & 2(t_2 - t_3) + 4\mathrm{i}\lambda & 0 \end{pmatrix}$$

The calculated eigenvalues are:

$$\varepsilon_1 = 2\sqrt{(t_2 - t_3)^2 + 4\lambda^2}, \varepsilon_2 = dE \text{ and } \varepsilon_3 = -2\sqrt{(t_2 - t_3)^2 + 4\lambda^2}$$

Therefore, the upper gap Δ_1 (the gap between top Dirac and middle flat bands) is determined by the onsite energy difference dE, the SOC strength λ and the geometric distortion $t_2 - t_3$.

$$\Delta_1(\mathrm{M1}) = dE - 2\sqrt{(t_2 - t_3)^2 + 4\lambda^2} \tag{5}$$

The critical energy for $\Delta_1 = 0$ is $dE_c = 2\sqrt{(t_2 - t_3)^2 + 4\lambda^2}$. For an ideal Lieb lattice ($t_2 - t_3 = 0$), the Δ_1 is solely determined by dE and λ.

Supplementary Note 4. Chern number and Berry curvature calculations.

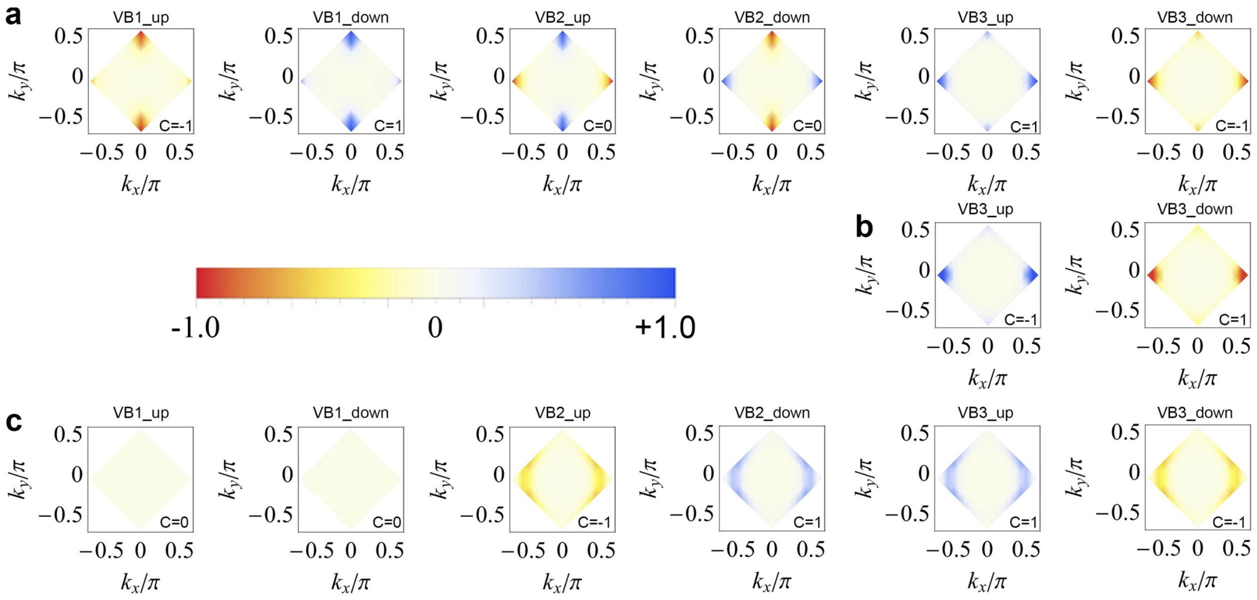

Supplementary Figure 2 | Berry curvature distributions corresponding to Figure 2 in the main text.

The Chern number for one isolated energy band can be calculated by the integral of the Berry curvature over the whole BZ

$$C_n = \frac{1}{2\pi} \int_{\mathrm{BZ}} \Omega_n(\mathbf{k}) d^2k. \tag{6}$$

The Berry curvature $\Omega_n(\mathbf{k})$ of the nth band can be determined as[3–5]

$$\Omega_n(\mathbf{k}) = -2\mathrm{Im}\sum_{m\neq n}\frac{\langle\psi_n(\mathbf{k})|v_x|\psi_m(\mathbf{k})\rangle\langle\psi_m(\mathbf{k})|v_y|\psi_n(\mathbf{k})\rangle}{(\varepsilon_{n\mathbf{k}}-\varepsilon_{m\mathbf{k}})^2} \quad (7)$$

where $\psi_n(\mathbf{k})$ and $\varepsilon_{n\mathbf{k}}$ are the spinor Bloch wave function and eigenvalue of the nth band at **k** point, and $v_{x(y)}$ is the velocity operator. The total Chern number[3] of a system is the sum of C_n over all the occupied bands (n):

$$C = \sum_n C_n = \frac{1}{2\pi}\sum_n \int_{\mathrm{BZ}} \Omega_n(\mathbf{k}) d^2k. \quad (8)$$

The topological properties of each band can be characterized by its spin Chern number, due to the spin degeneracy and conservation of S_z. The spin Chern number for the nth band is defined as $C_n^{\mathrm{s}} = (\mathrm{C}_{n\uparrow} - \mathrm{C}_{n\downarrow})/2$, and $C_{n\sigma}$ is Chern number for the spin-σ ($\sigma = \uparrow, \downarrow$) of the nth band, which can be calculated from the integral of the Berry curvature over the whole BZ[3,6] (see Supplementary Figure 2). As shown in Fig. 2 in the main text, when $dE = 0$, the calculated $C_1^{\mathrm{s}} = -1$ and $C_3^{\mathrm{s}} = +1$ for the top and bottom Dirac bands, respectively, and the calculated $C_2^{\mathrm{s}} = 0$ for middle flat band. Interestingly, when $dE > dE_{\mathrm{c}}$, the top Dirac and middle flat bands can switch their topologies, as shown in Fig. 2**c** in the main text, i.e., $C_1^{\mathrm{s}} = 0$ ($C_2^{\mathrm{s}} = -1$) for the top Dirac (middle flat) band.

Supplementary Note 5. Edge States Calculations.

The edge states can be obtained by calculating the edge density of states (DOS), $\rho(E, \mathbf{k}, \sigma) = (1/\pi)\mathrm{Tr}[\mathrm{Im}[g(E, \mathbf{k})]]_\sigma$, from the surface Green's function, $g(E, \mathbf{k})$[8]. By a truncation along Y-direction shown in the right panel of Supplementary Figure 1**b** to remove the periodicity along the X-direction, one can get a semi-infinite system terminated by an edge of corner sites (green), which reserves the translational symmetry along the Y-direction. The nontrivial topology of these Dirac-flat bands can be reflected by edge states calculations for a semi-infinite 2D Lieb lattice, as shown in Figs. 2**d**-2**f** in the main text. It can be seen that the helical edge states with opposite spin channels connect the two bands with nonzero C_n^{s}, indicating the quantum spin Hall effect. It is emphasized that in a realistic material, only the total spin Chern number C_{s}, the sum of C_n^{s} for all the occupied bands, is associated with the observable quantum conductance of the system. Therefore, the topological properties of a realistic Lieb lattice material depend on the position of Fermi level, and charge doping may be needed to achieve a nontrivial state.

Supplementary Note 6. Molecular-ligand-based Lieb lattice.

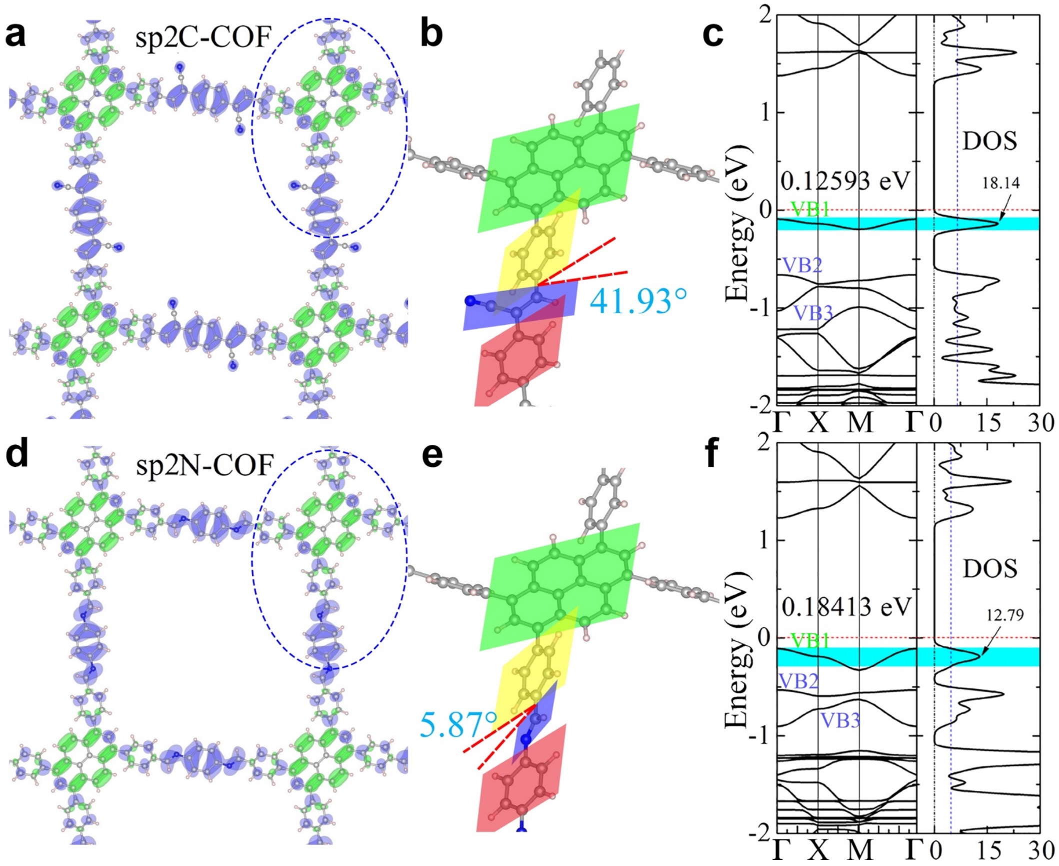

Supplementary Figure 3 | **(a)** Top view of monolayer *sp*^{2}C-COF with a distorted Lieb lattice structure, which includes the TPPy (BCNIB) ligands at the corner (edge) sites, with the plots of the partial charge distributions of the VB1, VB2 and VB3 in real space. **(b)** Zoom-in view of the local structure around the corner site, which is marked as the dashed ellipse in **(a)**. **(c)** Energy band structure and DOS of monolayer *sp*^{2}C-COF, where the narrow VB1 leads to a sharp peak of DOS (marked by light blue color) near the E_F. **(d)-(f)** Same to **(a)-(c)** but for *sp*^{2}N-COF, which are composed of the TPPy (corner) and BAIB (edge) ligands. The interfacial torsion angle τ between the edge and corner sites are 41.93° and 5.87° for *sp*^{2}C-COF and *sp*^{2}N-COF, respectively.

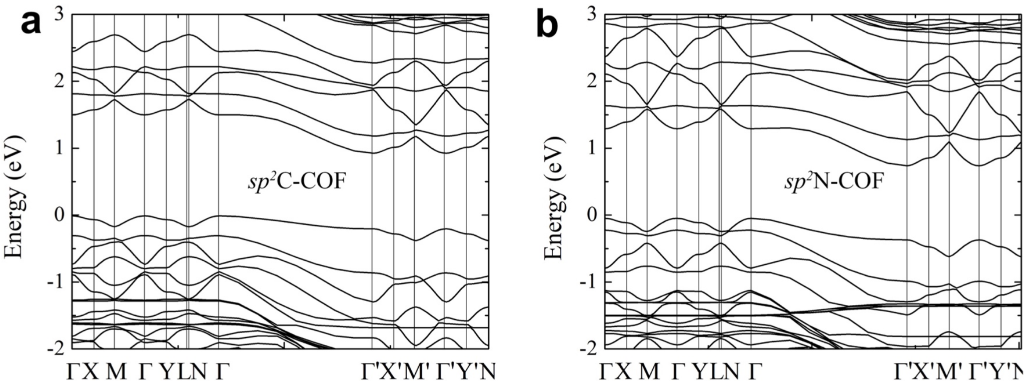

Supplementary Figure 4 | DFT-calculated band structures of **(a)** bulk sp^2C-COF and **(b)** bulk sp^2N-COF.

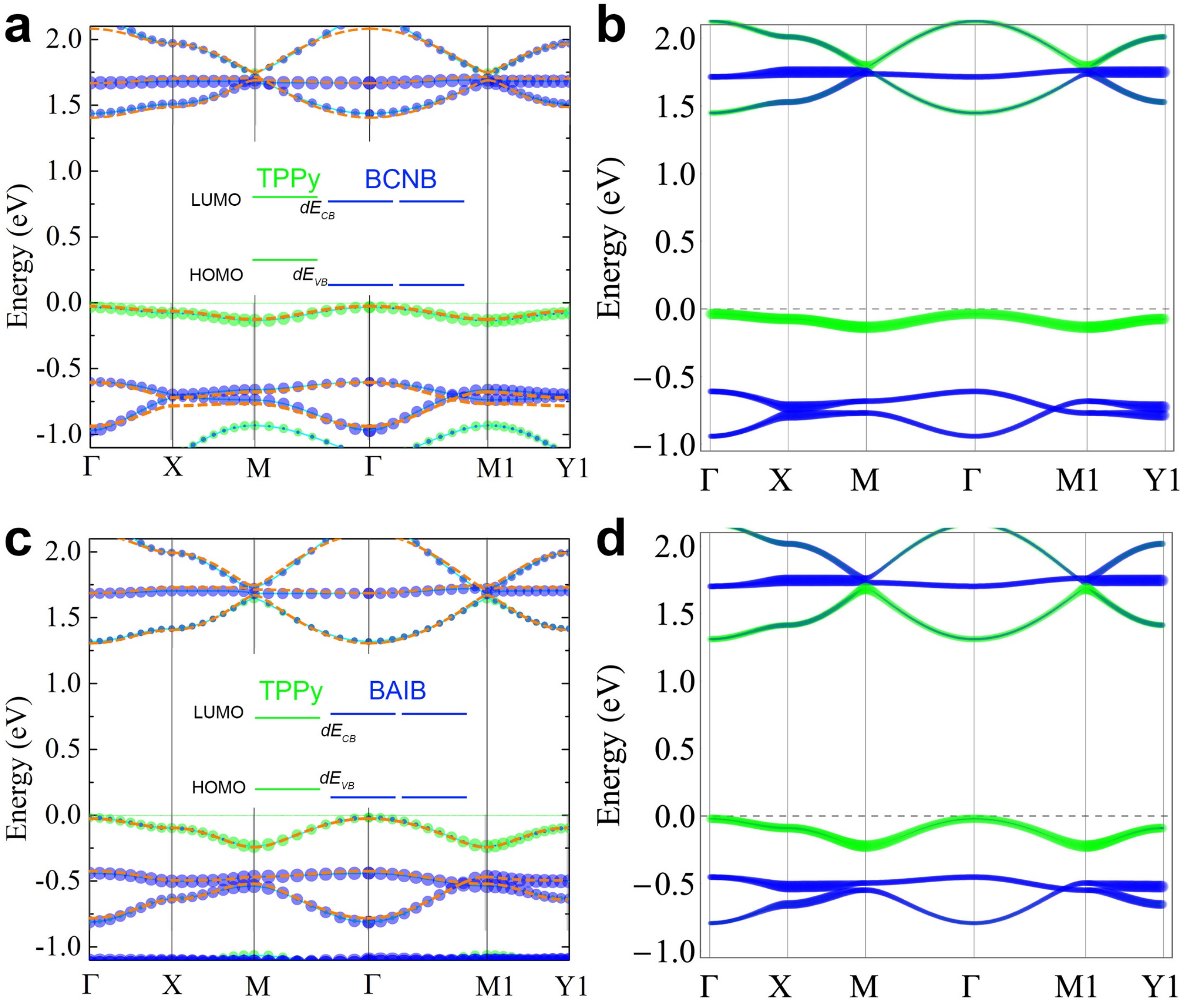

Supplementary Figure 5 | (a) and **(c)** six-band TB band fitting (orange-dashed lines) of the top three valence bands and bottom three conduction bands for the DFT-calculated *sp*^{2}C-COF and *sp*^{2}N-COF band structures, respectively. **(b)** and **(d)** Corresponding TB band structures with band projections for the *sp*^{2}C-COF and *sp*^{2}N-COF, respectively. Blue and green colors denote the contributions of the BCNB (BAIB) and TPPy in *sp*^{2}C-COF (*sp*^{2}N-COF), respectively. For *sp*^{2}C-COF: $t_{1\mathrm{H}} = t_1 = 0.1\mathrm{eV}$, $t_{2\mathrm{H}} = 0.4t_1 = 0.04\mathrm{eV}$, $t_{3\mathrm{H}} = 0.45t_{2\mathrm{H}} = 0.018\mathrm{eV}$, $dE_{\mathrm{H}} = 5.9t_1$, $\varepsilon_{1\mathrm{H}} = -0.135\mathrm{eV}$, $\varepsilon_{2\mathrm{H}} = \varepsilon_{3\mathrm{H}} = -0.725\mathrm{eV}$, $t_{1\mathrm{L}} = 1.2t_{1\mathrm{H}} = 0.12\mathrm{eV}$, $t_{2\mathrm{L}} = -0.1t_{1\mathrm{L}} = -0.04\mathrm{eV}$, $t_{3\mathrm{L}} = 0.45t_{2\mathrm{L}} = -0.018\mathrm{eV}$, $dE_L = 0.4t_{1\mathrm{L}}$, $\varepsilon_{1\mathrm{H}} = 1.79\mathrm{eV}$, and $\varepsilon_{2\mathrm{H}} = \varepsilon_{3\mathrm{H}} = 1.75\mathrm{eV}$. For *sp*^{2}N-COF: $t_{1\mathrm{H}} = t_1 = 0.12\mathrm{eV}$, $t_{2\mathrm{H}} = 0.4t_1 = 0.048\mathrm{eV}$, $t_{3\mathrm{H}} = 0.45t_{2\mathrm{H}} = 0.022\mathrm{eV}$, $dE_{\mathrm{H}} = 2.5t_1$, $\varepsilon_{1\mathrm{H}} = -0.222\mathrm{eV}$,$\varepsilon_{2\mathrm{H}} = \varepsilon_{3\mathrm{H}} = -0.522\mathrm{eV}$,$t_{1\mathrm{L}} = 1.25t_{1\mathrm{H}} = 0.15\mathrm{eV}$,$t_{2\mathrm{L}} = -0.1t_{1\mathrm{L}} = -0.015\mathrm{eV}$, $t_{3\mathrm{L}} = 0.45t_{2\mathrm{L}} = -0.007\mathrm{eV}$,$dE_L = 0.4t_{1\mathrm{L}}$,$\varepsilon_{1\mathrm{H}} = 1.686\mathrm{eV}$, and$\varepsilon_{2\mathrm{H}} = \varepsilon_{3\mathrm{H}} = 1.746\mathrm{eV}$. Insets of **(a)** and **(c)**: Band alignment for molecular orbitals (HOMOs and LUMOs) of the ligands.

Supplementary Note 7. Six-band tight-binding modeling.

$$H = \begin{pmatrix} H_{\mathrm{H}} & H_{\mathrm{HL}} \\ H_{\mathrm{HL}}^{\dagger} & H_{\mathrm{L}} \end{pmatrix} \tag{9}$$

A six-band TB Hamiltonian is employed to fit the top three VBs and bottom three CBs. The two diagonal blocks in Supplementary Equation 9 are the 3-orital Hamiltonians for HOMOs (H_{H}) and LUMOs (H_{L}), respectively.

$$H_{\mathrm{H/L}} = \begin{pmatrix} E_1 & E_{12} & E_{13} \\ E_{21} & E_2 & E_{32} \\ E_{31} & E_{32} & E_3 \end{pmatrix} \tag{10}$$

$E_1 = \varepsilon_{1\mathrm{H/L}}$ and $E_2 = E_3 = \varepsilon_{2\mathrm{H/L}}$. The NN hoppings are $E_{12} = -2t_{1\mathrm{H/L}}\cos(k_1 a/2)$, $E_{21} = E_{12}^*$, $E_{13} = -2t_{1\mathrm{H/L}}\cos(k_2 a/2)$ and $E_{31} = E_{13}^*$. The NNN hoppings are $E_{23} = -2t_{2\mathrm{H/L}}\cos[(-k_1 + k_2)\, a/2] - 2t_{3\mathrm{H/L}}\cos[(k_1 + k_2)\, a/2]$ and $E_{32} = E_{23}^*$.

H_{HL} and $H_{\mathrm{HL}}^{\dagger}$ are the interactions between the HOMOs and LUMOs. Due to the large band gaps of sp2C-COF and sp2N-COF, H_{HL} and $H_{\mathrm{HL}}^{\dagger}$ are neglected.

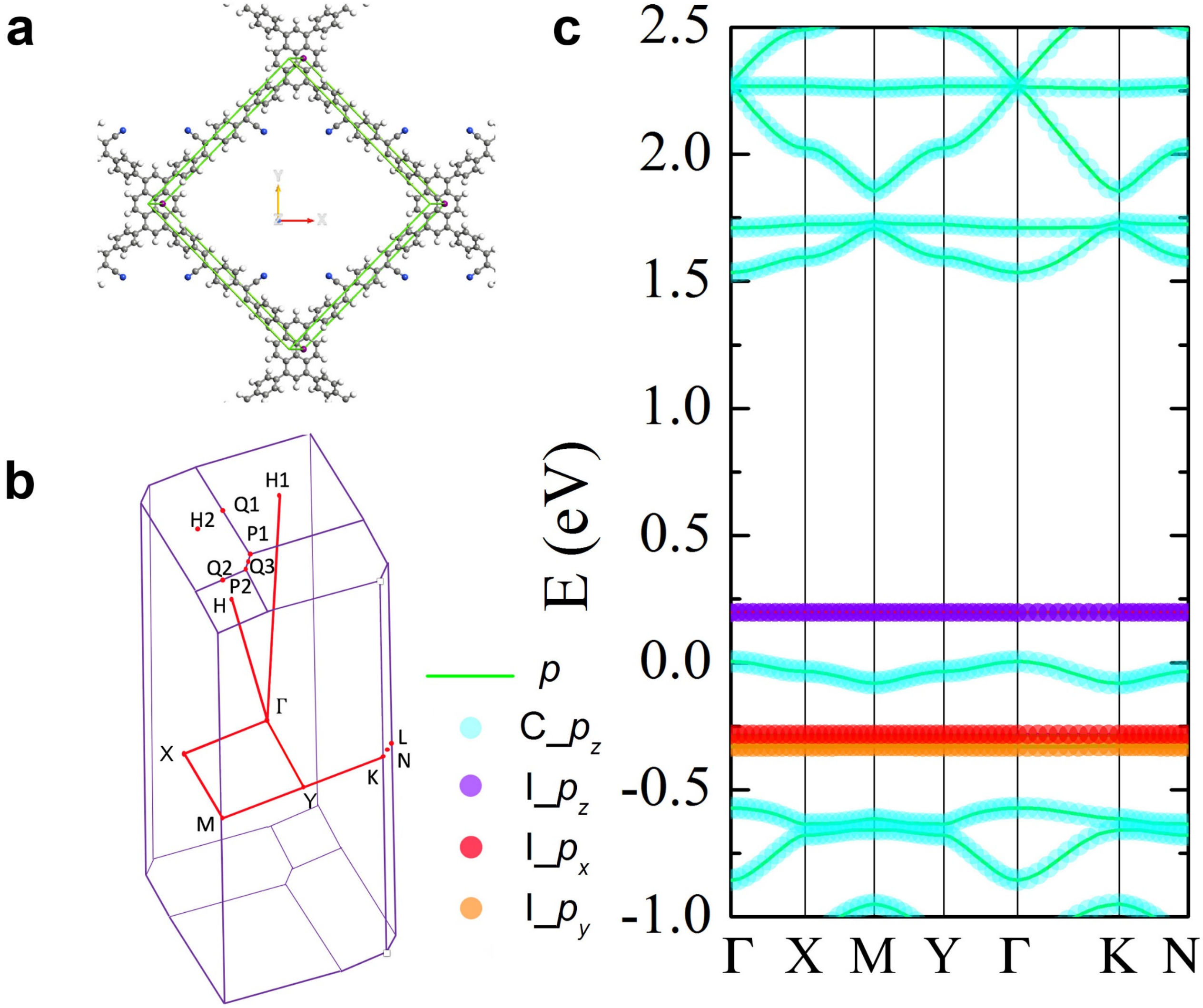

Supplementary Figure 6 | **(a)** Structure of iodine-doped *sp*^{2}C-COF. **(b)** BZ. **(c)** DFT-calculated projected band structure for iodine-doped *sp*^{2}C-COF. Circle sizes represent the weight of the orbitals. Cyan and blue colors denote the carbon orbitals on corner and edges, respectively. Grape, orange and pink colors depict the contribution of the iodine atom.

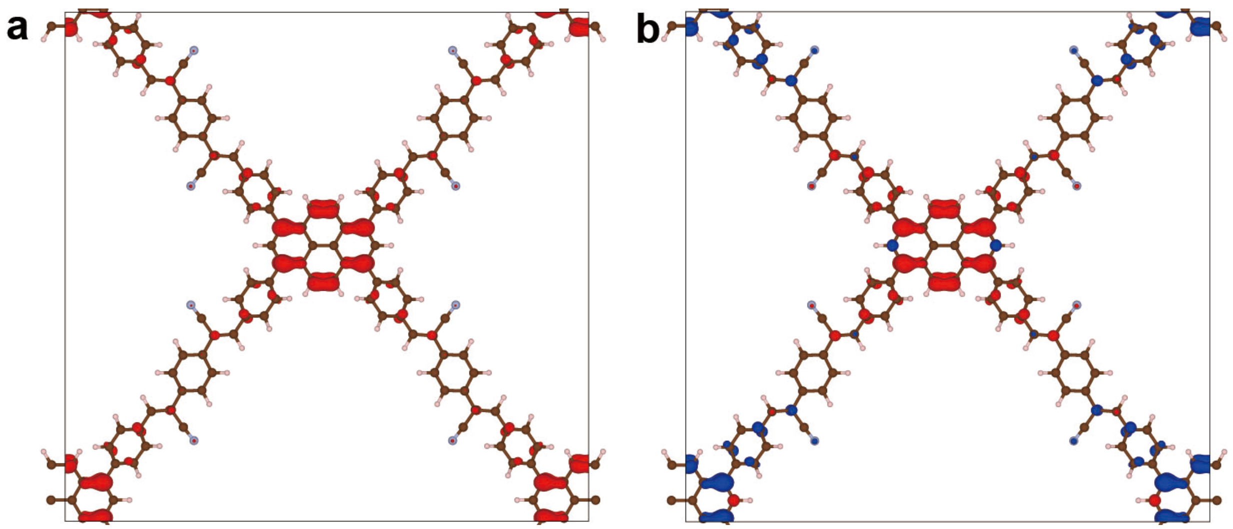

Supplementary Figure 7 | Spin density distributions of the *sp*[2]C-COF. DFT-calculated spin density distributions of *sp*[2]C-COF with one hole doping in a $\sqrt{2}\times\sqrt{2}$ supercell for **(a)** an FM state and **(b)** an AFM state.

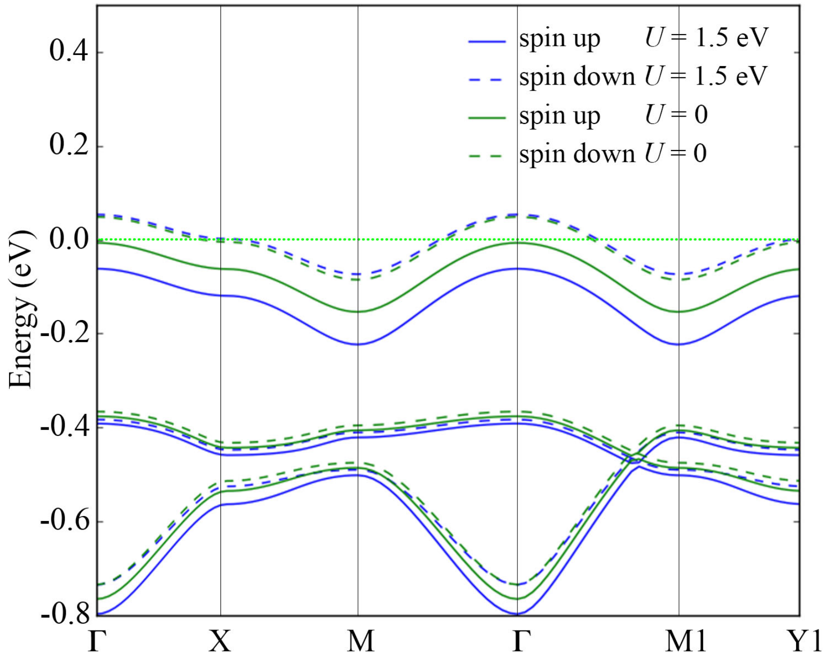

Supplementary Figure 8 | GGA+U calculated band structures with (U=1.5 eV) and without U.

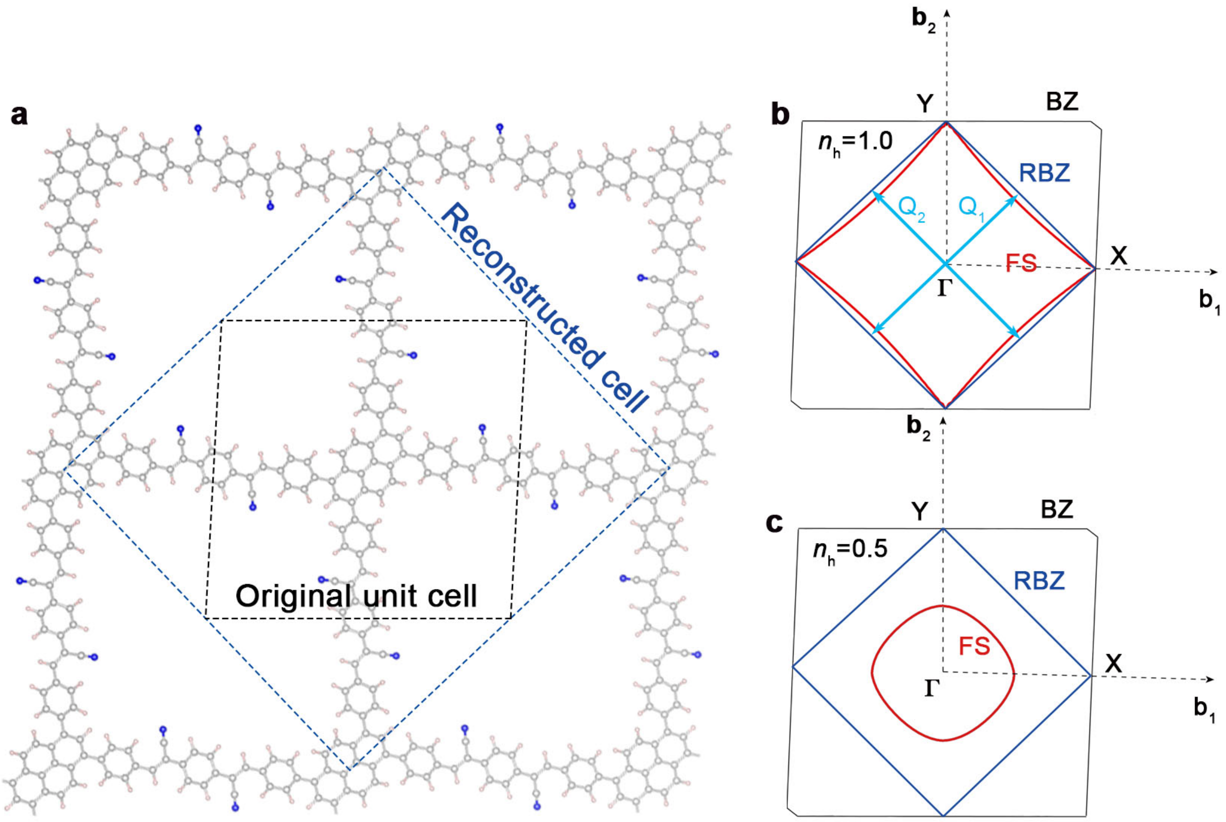

Supplementary Figure 9 | Mechanism of Fermi surfaces (FS) nesting under hole dopings. **(a)** Original unitcell and reconstructed supercell are marked by the black and blue dashed lines, respectively. The FS nesting for **(b)** n_h = 1.0 holes per u.c. and **(c)** n_h = 0.5 holes per u.c., respectively. The light blue arrows (Q1 and Q2) in **(b)** indicate the FS nesting vectors.

Supplementary Note 8. Half-metallic Semimetal.

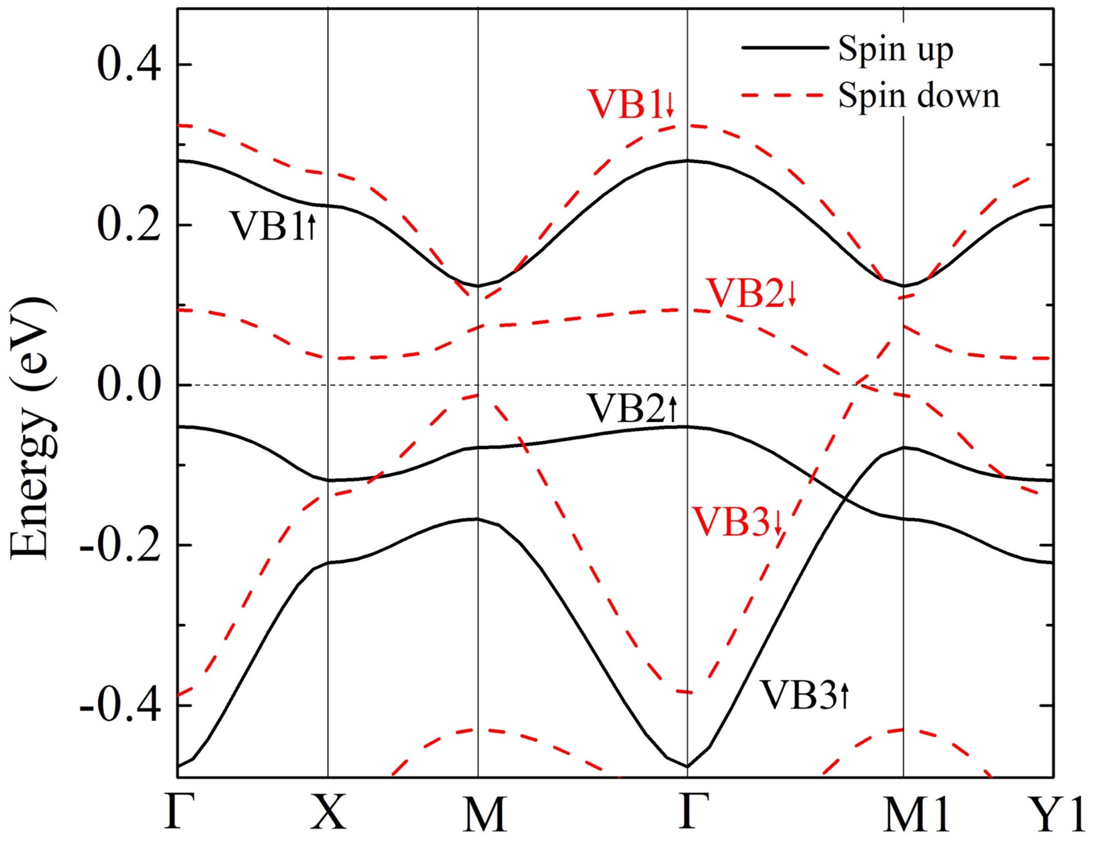

Supplementary Figure 10 | Half-Metallic Semimetal. DFT-calculated spin-polarized band structures of monolayer sp^2C-COF with three holes doping into each unitcell. E_{F} is set to zero.

Similar to VB1, VB2-VB3 also have relatively narrow band widths in sp^2C-COF and sp^2N-COF systems, as shown in Figs. 3**c** and 3**d** in the main text. Therefore, it is possible to realize a half-metallic semimetal when VB2 is partially occupied with spontaneous spin polarization. To test our intuition, we dope three extra holes into one unitcell of the sp^2C-COF system, which corresponding to a hole concentration of $\sim 4.9 \times 10^{13}$ cm^{-2}. This might be achieved in the experiments by heavy iodine doping or gating doping[7–9]. As shown in Supplementary Figure 10, our calculations indeed show that the Dirac cone can be fully spin-polarized, giving rise to a novel half-metallic phase. The half-metallic gap is estimated to be as large as 0.1 eV in sp^2C-COF.

Supplementary Table 1. Hole doping induced spin polarization in *sp*^{2}C-COF and *sp*^{2}N-COF (0.5 hole per unit cell).

COF	NM (eV)	FM (eV)	AFM (eV)	Magnetization energy (meV)	Magnetic moment (μ_B per hole)
***sp*^{2}C-COF**	-1456.4279	-1456.4348	-1456.4298	-6.9	1.0000
***sp*^{2}N-COF**	-1317.7573	-1317.7598	-1317.7573	-1.5	0.7761

Supplementary References:

1. Wang, Y. F., Gu, Z. C., Gong, C. De & Sheng, D. N. Fractional quantum Hall effect of hard-core bosons in topological flat bands. *Phys. Rev. Lett.* **107**, 146803 (2011).
2. Kane, C. L. & Mele, E. J. Quantum Spin Hall Effect in Graphene. *Phys. Rev. Lett.* **95**, 226801 (2005).
3. Thouless, D. J., Kohmoto, M., Nightingale, M. P. & den Nijs, M. Quantized Hall Conductance in a Two-Dimensional Periodic Potential. *Phys. Rev. Lett.* **49**, 405–408 (1982).
4. Fang, Z. The Anomalous Hall Effect and Magnetic Monopoles in Momentum Space. *Science* **302**, 92–95 (2003).
5. Yao, Y. *et al.* First Principles Calculation of Anomalous Hall Conductivity in Ferromagnetic bcc Fe. *Phys. Rev. Lett.* **92**, 037204 (2004).
6. Fukui, T., Hatsugai, Y. & Suzuki, H. Chern numbers in discretized Brillouin zone: Efficient method of computing (spin) Hall conductances. *J. Phys. Soc. Japan* **74**, 1674–1677 (2005).
7. Deng, Y. *et al.* Gate-tunable room-temperature ferromagnetism in two-dimensional Fe3GeTe2. *Nature* **563**, 94–99 (2018).
8. Efetov, D. K. & Kim, P. Controlling Electron-Phonon Interactions in Graphene at Ultrahigh Carrier Densities. *Phys. Rev. Lett.* **105**, 256805 (2010).
9. Ye, J. T. *et al.* Superconducting Dome in a Gate-Tuned Band Insulator. *Science* **338**, 1193–1196 (2012).
